# Synergistic effects of the curcumin analog HO-3867 and olaparib in transforming fallopian tube epithelial cells

**DOI:** 10.1007/s10637-025-01571-2

**Published:** 2025-08-04

**Authors:** Cai-Chieh Tseng, Min-Hsi Ku, Wei-Min Wu, Ava Mendez, Tessa Christner, Yun-Chieh Wu, Wei-Lun Huang, Yu-Hsiang Chen, Ching-Wen Huang, Johnathan Barefoot, Chi-Wei Chen

**Affiliations:** 1 Department of Biochemical and Molecular Medical Sciences, College of Science and Engineering, National Dong Hwa University, Hualien, 974301 Taiwan, R.O.C.; 2 Department of Natural Resources and Environmental Studies, College of Environmental Studies, National Dong Hwa University, Hualien, 974301 Taiwan, R.O.C.; 3 Department of Biology, College of Arts and Sciences, Appalachian State University, Boone, NC 28608 USA

**Keywords:** HO-3867, Curcumin analog, PARP inhibitor, *TP53*, Transforming fallopian tube epithelial cells, Ovarian cancer

## Abstract

Ovarian cancer remains one of the most lethal gynecologic malignancies, largely due to high recurrence rates and treatment-related toxicities. Although PARP inhibitors like Olaparib have shown efficacy in BRCA-mutated cancers, their benefit is limited in broader patient populations. *TP53* mutations, highly prevalent in ovarian cancer, promote tumor progression and resistance, making p53 a key therapeutic target. This study evaluated the anticancer potential of HO-3867, a curcumin analog known to restore mutant p53 function, alone and in combination with Olaparib. We used fallopian tube-derived ovarian cancer models harboring mutant or null *TP53* and analyzed *TP53* expression and mutation profiles using TCGA datasets. Molecular docking simulations and cellular thermal shift assays (CETSA) confirmed HO-3867 binding to the p53^Y220C^ mutant core domain. Cytotoxicity was assessed via SRB assays; flow cytometry and Western blotting were used to examine cell cycle progression, apoptosis, and DNA damage. HO-3867 treatment increased phospho-p53 (Ser15) and p21 expression, induced G1 phase arrest, and suppressed cell viability. Notably, co-treatment with Olaparib synergistically enhanced apoptosis, as indicated by increased caspase-3 and PARP1 cleavage and elevated γH2AX levels. These findings suggest that HO-3867 reactivates mutant p53 and potentiates Olaparib efficacy by promoting apoptosis and amplifying DNA damage, offering a promising therapeutic strategy for *TP53*-mutant ovarian cancer.

## Introduction

Ovarian cancer is one of the most prevalent and lethal gynecological malignancies, accounting for the highest mortality rate among reproductive system cancers in women [1, 2]. Despite advancements in surgery, radiotherapy, and chemotherapy, the prognosis remains poor due to high recurrence rates, metastasis, and the development of resistance to standard therapies [3]. While PARP inhibitors have shown therapeutic promise, their efficacy is confined mainly to patients with BRCA mutations, leaving a substantial proportion of cases without effective targeted treatment options [4]. This underscores the urgent need for innovative therapeutic strategies that can address the broader ovarian cancer patient population. Given its aggressive nature, poor clinical outcomes, and limited treatment options, ovarian cancer represents a critical and unmet medical challenge that demands the development of more effective and inclusive therapeutic approaches.

The *TP53* gene, frequently mutated in ovarian cancer, especially in high-grade serous ovarian carcinoma (HGSOC), encodes a protein that regulates cell division and prevents uncontrolled cell growth [5]. The earliest detectable transformation of the fallopian tube fimbrial epithelium (FTE) is lesions involving *TP53* mutations, characterized by an accumulation of mutant p53 protein in a series of secretory cells referred to as the"p53 signature"[6, 7]. *TP53* mutations are the most common genetic alterations in sporadic epithelial ovarian cancer (EOC), with most HGSOCs harboring inactive p53 due to single-point mutations [5]. This underscores the critical role of p53 in suppressing ovarian tumor development and progression.

Mutations in *TP53* impair the tumor-suppressive functions and confer oncogenic properties, driving cellular transformation, tumor progression, and resistance to chemotherapy [8]. Restoring p53 activity has shown potential in preclinical models, where reactivation of p53 inhibited tumor growth in mice [8–11]. The curcumin analog HO-3867, a bifunctional compound with a diarylidenyl-piperidone (DAP) backbone conjugated to an N-hydroxypyrroline (–NOH) group, has emerged as a promising candidate for ovarian cancer therapy [12]. Notably, it reactivates mutant p53 by converting it to a wild-type conformation, inducing tumor-suppressive responses and rescuing the suppression of PLAC1 expression in ovarian cancer cells [13]. Interestingly, HO-3867 has been identified as a potent p53 restorer, capable of reactivating mutant p53 through covalent binding. This reactivation triggers a wild-type p53-like anticancer response, marked by selective cytotoxicity toward cancer cells and strong efficacy in tumor models [14]. These properties position HO-3867 as a highly promising therapeutic candidate with significant clinical potential. Preclinical studies have shown that HO-3867 suppresses tumor growth, inhibits metastasis, and maintains a favorable safety profile, making it a compelling candidate for ovarian cancer treatment.

In this study, we explored the therapeutic potential of the p53 restorer HO-3867, both as a monotherapy and in combination with the PARP inhibitor Olaparib, to overcome the limitations of current ovarian cancer treatments. HO-3867's selective inhibition of mutant p53 and ability to induce apoptosis in ovarian cancer cells make it a promising agent for targeting a broader patient population. As HGSOC is believed to originate from the fallopian tube epithelium, we utilized human transforming fallopian tube epithelial (TFTE) cells as a model system, including FT282-CCNE1 (p53^R175H^) and FE25 (p53 null) cells [15–17]. To further validate the therapeutic potential of combining HO-3867 with Olaparib, we extended the synergy analysis to HGSOC cell lines OVCAR8, which carries a p53 Y126 to K132 deletion, and PEO4, which harbors a p53 G244D mutation. This approach provides a biologically relevant platform to study the molecular mechanisms underlying HGSOC development and progression and evaluate potential therapeutic interventions in a context that closely mimics the tumor's tissue of origin [18–20]. Our results demonstrate that HO-3867 synergizes with Olaparib to enhance therapeutic efficacy, inhibiting cancer cell proliferation through mechanisms including G1 cell cycle arrest, induction of apoptosis, and elevated DNA double-strand breaks, as evidenced by increased γH2AX levels. These findings elucidate the molecular basis of HO-3867’s anticancer effects and its synergy with PARP inhibition, highlighting its potential as a complementary therapeutic strategy to improve clinical outcomes in ovarian cancer patients.

## Materials and methods

### Materials

Cell culture reagents, including Roswell Park Memorial Institute 1640 medium (RPMI-1640), fetal bovine serum (FBS), and penicillin/streptomycin, were purchased from Gibco-BRL (Gaithersburg, MD, USA). HO-3867 (HY-100453), Olaparib (HY-10162), and MG-132 (HY-13259) were obtained from MedChemExpress (Monmouth Junction, NJ, USA). Primary antibodies against γH2AX (Ser139, 05–636) and vinculin (MAB3574) were purchased from Merck Millipore (Billerica, MA, USA). Antibodies for phospho-p53 (Ser15, 82530), total p53 (2527), and p21 (2947) were obtained from Proteintech (Rosemont, IL, USA). Antibodies against caspase-3 (A19654) and PARP1 (A0942) were acquired from Abclonal (Woburn, MA, USA). The antibody targeting Actin (8457 T) was purchased from Cell Signaling (Danvers, MA, USA). The CoraLite® 488 Annexin V/PI Apoptosis Detection Kit (PF00005) was also sourced from Proteintech. Unless otherwise specified, all other chemicals and reagents were purchased from Sigma-Aldrich (St. Louis, MO, USA).

### TCGA database

The TCGA PanCancer Atlas data were accessed and retrieved from cBioPortal (https://www.cbioportal.org/) [21, 22]. Kaplan–Meier curves depicting 5-year overall survival rates based on wildtype and mutant TP53 expression were obtained from KMplot (https://kmplot.com/analysis/). It integrates gene expression and clinical outcome data from multiple publicly available datasets, including The Cancer Genome Atlas (TCGA), Gene Expression Omnibus (GEO), and the European Genome-phenome Archive (EGA). The tool enables stratification of patients based on gene expression levels to assess their association with overall survival. Median expression was used as the cutoff for grouping, and log-rank p-values were calculated to determine statistical significance [23, 24]. *TP53* expression data were profiled using TCGA and GTEx databases via GEPIA2 (http://gepia2.cancer-pku.cn/) [25] and ULACAN (https://ualcan.path.uab.edu/index.html) [26], with transcript per million (TPM) used as the expression unit. Statistical significance is indicated as *p* < 0.001 compared to normal tissues. Additional p53 protein expression data were retrieved from the Protein Atlas (https://www.proteinatlas.org/) [27].

### In silico docking

Virtual protein docking was conducted using the online platform DockingServer (www.dockingserver.com) [28, 29]. The structure of the p53 cancer mutant Y220C protein was retrieved from the Protein Data Bank (PDB; ID: 5O1A) [30]. Energy minimization of the ligand molecule, HO-3867, was carried out using the Merck Molecular Force Field 94 [31]. For docking simulations, Gasteiger partial charges, non-polar hydrogen atoms, rotatable bonds, essential hydrogen atoms, Kollman united atom type charges, affinity grid, and solvation parameters were applied through AutoDock tools [32]. The van der Waals and electrostatic terms were calculated using AutoDock’s parameter-dependent and distance-dependent dielectric functions. The docking simulations employed the Lamarckian genetic algorithm combined with the Solis & Wets local search method [33], with each experiment consisting of 10 independent runs to ensure robust and reliable results.

### Cellular thermal shift assay (CETSA)

To evaluate the interaction between HO-3867 and p53, CETSA [34, 35] was performed in FT282-CCNE1 cells harboring the P53^R175H^ mutation. Cells were first pre-treated overnight with 10 µM MG-132 to inhibit proteasomal degradation. The following day, cells were incubated with 10 µM HO-3867 for 3 h. Treated cells were then harvested, resuspended in phosphate-buffered saline (PBS), and aliquoted into six 50 µL tubes. Cell suspensions were subjected to thermal challenge at 25.0 °C, 43.3 °C, 45.1 °C, 46.7 °C, 48.3 °C, or 49.7 °C for 5 min. Subsequently, cells were lysed using three cycles of freeze–thaw between − 80 °C and 37 °C. The lysates were centrifuged at 13,000 × g for 10 min at 4 °C to remove cellular debris. Supernatants (35 µL) were collected, mixed with SDS sample buffer, and boiled at 105 °C for 5 min. Protein stability and thermal shifts were analyzed by Western blotting using antibodies against p53 and vinculin.

### Cell culture conditions

FT282-CCNE1 cells were generously provided by Dr. Ronny Drapkin (University of Pennsylvania) [15], and FE25 cells were kindly provided by Dr. Tang-Yuan Chu (Tzu Chi University) [16]. Both cell lines, derived from transforming fallopian tube epithelial cells. OVCAR8 and PEO4 cells were kindly provided by Dr. Katherine Aird (University of Pittsburgh). All lines were cultured in RPMI 1640 medium supplemented with 10% fetal bovine serum (FBS) and 1% penicillin (100 U/mL)/streptomycin (100 μg/mL). The cultures were maintained at 37 °C in a humidified atmosphere with 5% CO₂.

### In vitro cell viability assay

Exponentially proliferating cells were treated with various compounds at concentrations ranging from 0.41 to 100 µM for 5 days. HO-3867 and Olaparib were initially dissolved in 100% DMSO to prepare a 20 mM stock solution of HO-3867. Appropriate amounts of the stock solution were diluted into the culture medium to achieve the desired final concentrations. A threefold serial dilution was applied. The final concentration of DMSO in the medium was maintained at 0.05% (v/v), a level previously shown to be non-toxic to cells [36], and 0.5% DMSO (without HO-3867) was used as a blank control [37–43]. Cell viability was assessed using the sulforhodamine B (SRB) assay as in previous studies [44], and absorbance was measured at 510 nm using a plate reader. The proliferation rate was calculated following the manufacturer’s instructions. The half-maximal inhibitory concentration (IC50) and 25% inhibition concentration (IC_25_) of each compound were determined by analyzing dose–effect relationships using CompuSyn software [45]. Doubling times for cell proliferation were calculated and monitored over the 5-day treatment period.

### Drug combination studies

To assess the combinatorial effects of HO-3867 and Olaparib, FT282-CCNE1 and FE25 cells were treated with both agents using either simultaneous or sequential administration. For the primary combination assay, HO-3867 was used at a fixed concentration corresponding to its IC₂₅ value (determined from single-agent dose–response curves), while Olaparib was applied at increasing concentrations. For simultaneous treatment, both drugs were added concurrently and incubated for 5 days under standard culture conditions. For sequential treatments, Olaparib was added either 4 h before or 4 h after the addition of HO-3867, followed by continued incubation for 5 days. Cell viability was assessed using the sulforhodamine B (SRB) assay. Combination index (CI) values were calculated using the Chou–Talalay method implemented in CompuSyn software to evaluate drug interactions, where CI < 1 indicates synergy, CI = 1 indicates additivity, and CI > 1 indicates antagonism [45].

### Colony formation

FT282-CCNE1 cells (5 × 10^3^ per well) were seeded in 24-well plates in 250 μL of RPMI-1640 medium supplemented with 10% FBS and allowed to adhere overnight. The following day, cells were exposed to 4.5 μM HO-3867 or 16 μM Olaparib in RPMI-1640 supplemented with 10% FBS for 14 days. For combination treatments, HO-3867 and Olaparib were administered either simultaneously or sequentially, with Olaparib given 4 h before or after HO-3867. Colony formation was assessed by fixing the cells in 1% paraformaldehyde for 5 min and staining with 0.05% crystal violet for 20 min. Excess stain was removed by destaining in 500 mL of 10% acetic acid for 5 min. The absorbance at 590 nm was measured using a Spectra Max 190 spectrophotometer (Molecular Devices, San Jose, CA, USA). All treatments were performed in triplicate for statistical reliability.

### Flow cytometric analysis

The effect of HO-3867 and Olaparib on the cell cycle distribution of FT282-CCNE1 cells was assessed by measuring cellular DNA content and apoptosis using flow cytometry, as previously described [46]. Following the treatments, cells were stained with propidium iodide (PI) and prepared in a single 15 mL conical tube for analysis. DNA content was quantified using a BD Accuri™ C6 Plus personal flow cytometer, providing precise insights into cell cycle phases and apoptotic populations. Data analysis was further refined using BD Accuri C6 software to ensure accurate interpretation of the results (BD Biosciences, San Jose, CA, USA).

### Annexin V-FITC apoptosis staining assay

The pro-apoptotic effect of HO-3867 on FT282-CCNE1 cells was evaluated by measuring cellular DNA content and apoptosis through flow cytometry, as previously described [47]. After treatment, apoptosis was assessed using the FITC Annexin V Apoptosis Detection Kit I, following the manufacturer’s instructions. The CoraLite® 488-Annexin V and PI Apoptosis Kit (PF00005) was procured from Proteintech (Rosemont, IL, USA). Data acquisition was performed on a BD Accuri™ C6 Plus personal flow cytometer, and detailed analysis was carried out using BD Accuri C6 software (BD Biosciences, San Jose, CA, USA) for precise interpretation of the results [38].

### Protein extraction and western blot analysis

To explore the molecular mechanisms in greater detail, initiator and effector caspases, along with associated signaling pathways, were analyzed through Western blotting. Approximately 1.0 × 10⁶ FT282-CCNE1 cells per dish were cultured in 10 cm dishes and treated with HO-3867 and Olaparib for 3 h or 5 days as indicated. Total cell lysates were collected using SDS-containing sample buffer and denatured by boiling at 105 °C. Western blot analysis used specific primary antibodies against p21, phospho-p53, p53, γH2AX (Ser139), pro-caspase 3, cleaved caspase 3, pro-PARP1, and cleaved PARP1. After incubation with primary antibodies, the blots were treated with horseradish peroxidase-conjugated goat anti-rabbit or anti-mouse secondary antibodies to detect protein expression.

### Statistical analysis

All statistical analyses were conducted using one-way analysis of variance (ANOVA) to evaluate differences among treatment groups. Tukey's post hoc test was applied for multiple comparisons in groups with equal sample sizes. Data were analyzed using GraphPad Prism software, version 5.01. Results are presented as mean ± standard error of the mean (SEM), and p values less than 0.05 were considered statistically significant.

## Results

### Mutations and expression of TP53 in ovarian cancer

**Fig. 1:**
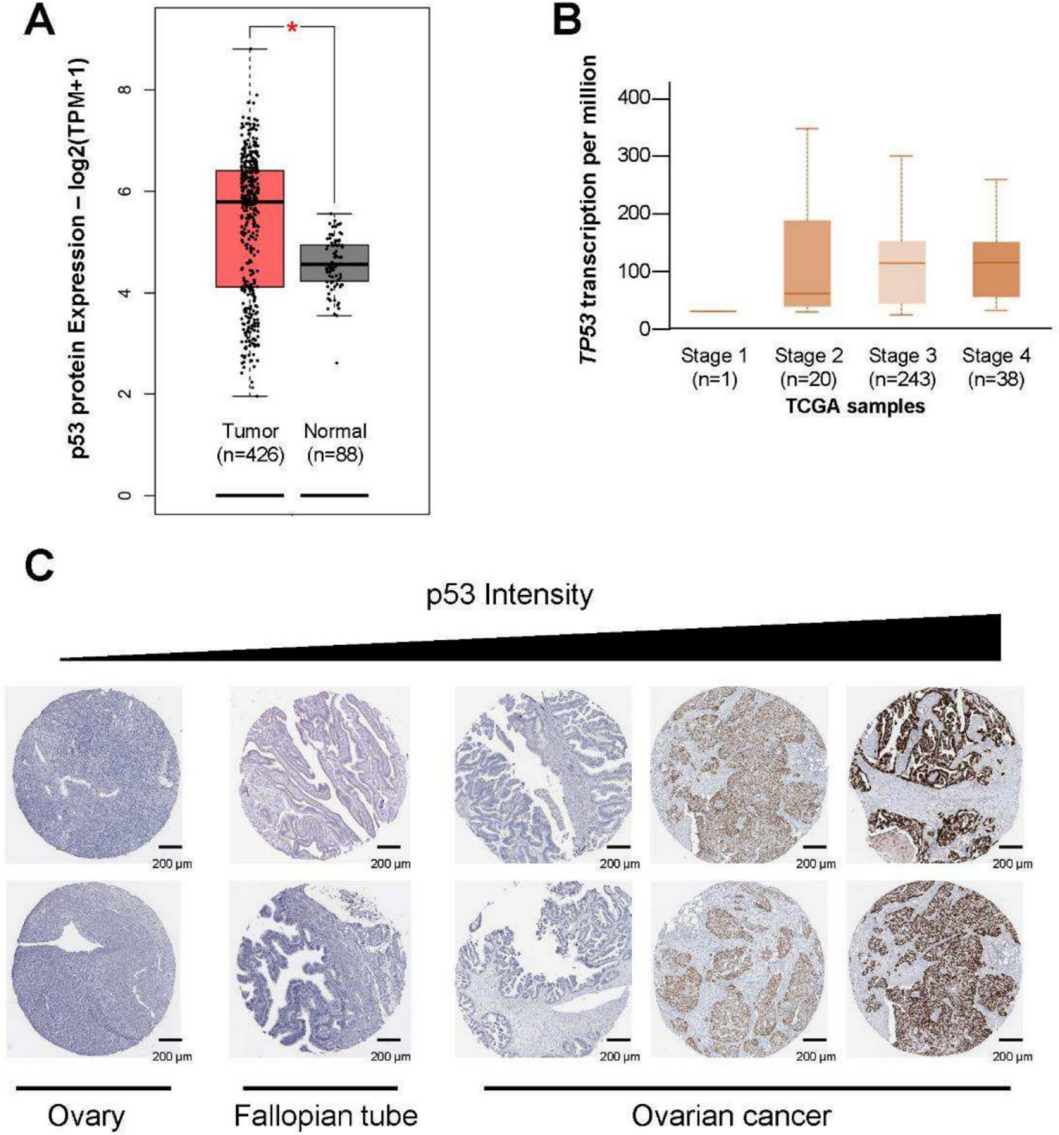
Upregulation of *TP53* in ovarian cancer patients. **A** Total p53 protein expression levels in normal fallopian tube tissues compared to high-grade serous ovarian carcinoma (HGSOC) specimens using GEPIA 2. The expression log_2_(TPM + 1) represents a mathematical transformation of Transcripts Per Million (TPM) values. Statistical significance is indicated as follows: *p < 0.05. **B** Boxplot analysis of TP53 mRNA expression levels across normal and tumor tissues in TCGA PanCancer Atlas studies, performed using UALCAN (http://ualcan.path.uab.edu). Tumor samples are stratified by *TP53* mutation status. Expression is shown as transcripts per million (TPM), and statistical comparisons highlight elevated *TP53* expression in tumors with TP53 mutations compared to normal tissues and *TP53* wild-type tumors. **C** P53 protein expression in normal fallopian tube tissues and HGSOC specimens, assessed by immunohistochemistry (IHC) using images from the Human Protein Atlas (https://www.proteinatlas.org). Representative IHC images show low or undetectable P53 staining in normal tissues, with progressively stronger nuclear staining observed in HGSOC samples, consistent with P53 overexpression or stabilization due to mutation. Scale bars: 200 µm

*TP53,* a key tumor suppressor gene, regulates cell proliferation and apoptosis [48]. Elevated nuclear p53 expression has improved outcomes in epithelial ovarian cancer. As shown in Fig. 1 A, p53 protein expression is significantly higher in ovarian cancer tissues compared to normal tissues. Notably, p53 protein levels exhibit a pronounced increase across individual stages of ovarian cancer progression (Fig. 1B). Data from The Human Protein Atlas further support these findings, demonstrating progressively elevated p53 expression levels in ovarian cancer tissues, as assessed by immunohistochemistry (IHC) (Fig. 1C), ranging from low to high expression. Together, these results underscore the critical role of p53 activity in ovarian cancer progression and highlight its potential implications for disease characterization and therapeutic targeting.

**Fig. 2:**
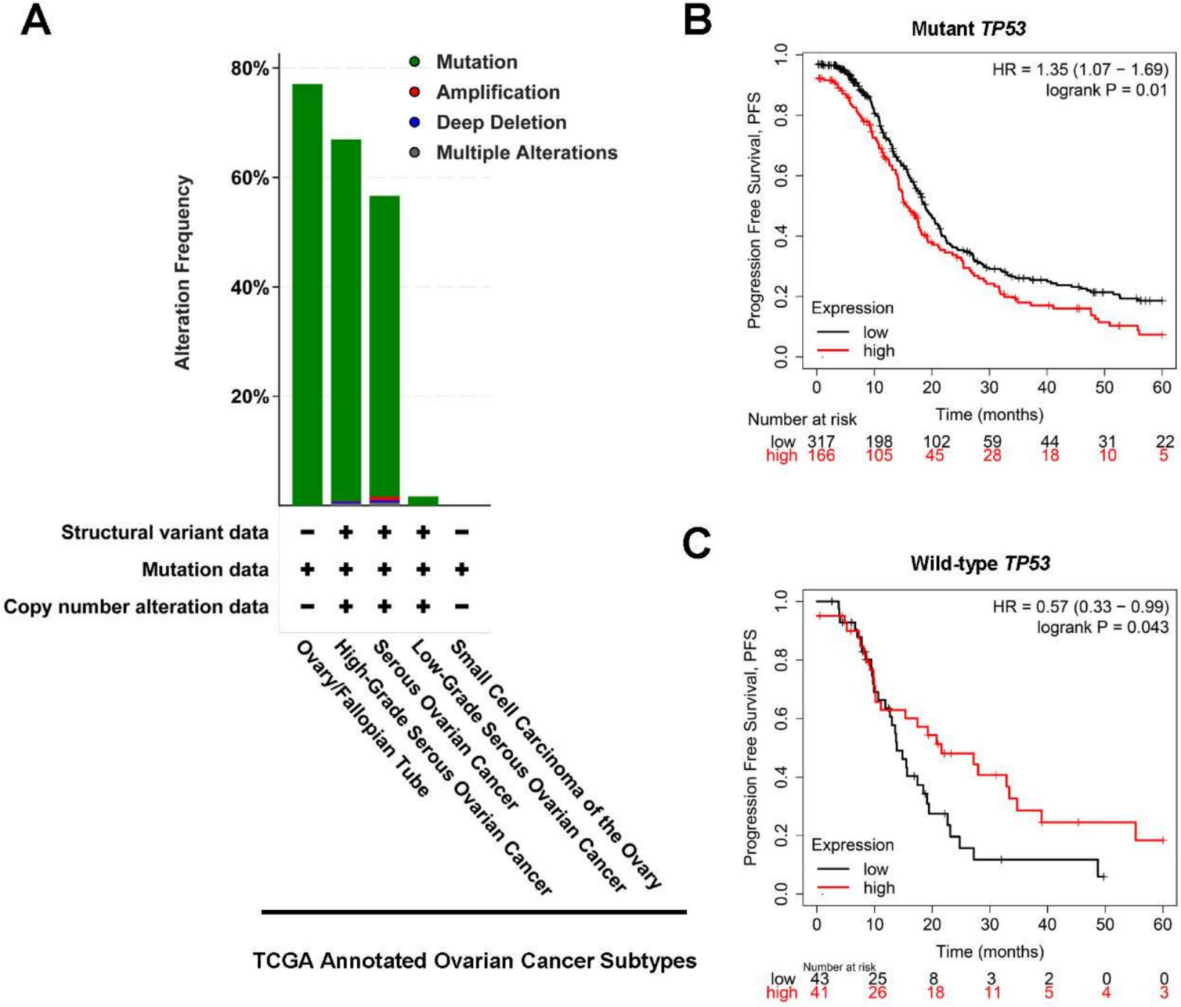
High mutation rate of *TP53* in ovarian cancer patients. **A** Analysis of *TP53* alterations across ovarian cancer subtypes based on TCGA “Cancer Type Detailed” annotations using cBioPortal. The high alteration frequency observed in the group labeled “Ovary/Fallopian Tube” corresponds to high-grade serous ovarian carcinoma (HGSOC), which originates from the fallopian tube epithelium and is classified under this category in the TCGA dataset. Alteration types include mutation (green), amplification (red), deep deletion (blue), and multiple alterations (gray). Note that these labels represent annotated cancer subtypes, not non-cancerous tissues. **B** Kaplan–Meier survival analysis of 5-year progression-free survival (PFS) in epithelial ovarian cancer (EOC) patients with mutant *TP53*, stratified by *TP53* expression levels. Survival curves were generated using the KM Plotter tool. Patients with high TP53 expression (red line) exhibited significantly shorter PFS compared to those with low expression (black line), with a hazard ratio (HR) of 1.35 (95% CI: 1.07–1.69) and a log-rank p value of 0.01. **C** Kaplan–Meier survival analysis of 5-year progression-free survival (PFS) in epithelial ovarian cancer (EOC) patients with wild-type *TP53*, stratified by *TP53* expression levels. Survival curves were generated using the KM Plotter tool. Patients with high *TP53* expression (red line) showed significantly improved PFS compared to those with low expression (black line), with a hazard ratio (HR) of 0.57 (95% CI: 0.33–0.99) and a log-rank *p* value of 0.043

Mutations in *TP53* are strongly associated with tumorigenesis, with somatic mutations in this gene commonly observed in ovarian cancer occurring in nearly half of ovarian tumors [49]. Analysis of The Cancer Genome Atlas (TCGA; cbioportal.com) data reveals significant variability in the prevalence of *TP53* mutations across different tumor types, affecting over 50% of patients (Fig. 2A). In ovarian cancer, *TP53* mutations have been linked to improved response rates to platinum-based therapy combined with chemotherapy, suggesting its potential as a critical biomarker for treatment response [50]. We investigated whether *TP53* expression, whether wild-type or mutant, is associated with progression-free survival (PFS) in ovarian cancer patients. Kaplan–Meier survival analysis was performed using the KM Plotter tool (https://kmplot.com/analysis/), which integrates gene expression and clinical outcome data from The Cancer Genome Atlas (TCGA), Gene Expression Omnibus (GEO), and the European Genome-phenome Archive (EGA) [23, 24]. The results showed that patients with high levels of mutant *TP53* expression exhibited significantly lower PFS, whereas those with high expression of wild-type *TP53* had improved PFS (Fig. 2B-C). These findings suggest that restoring mutant *TP53* to its wild-type form may improve clinical outcomes in ovarian cancer.

### Cytotoxicity of HO-3867 in human transforming fallopian tube epithelial cells

**Fig. 3:**
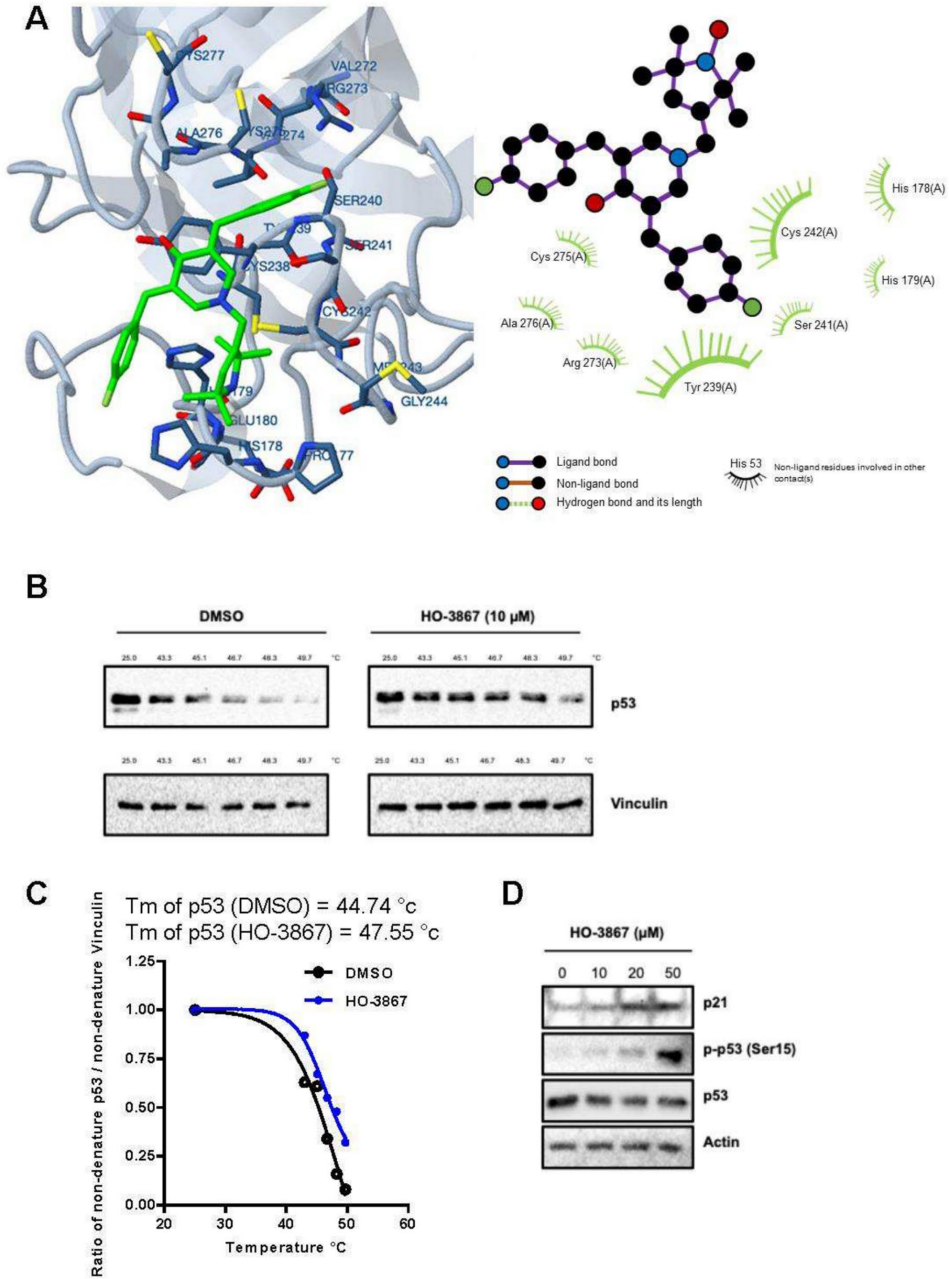
HO-3867 binds and functionally modulates mutant p53 in ovarian cancer cells. **A** Molecular docking analysis showing the predicted binding of HO-3867 to the core domain of the p53^Y220C^ cancer mutant. **B** Cellular thermal shift assay (CETSA) assessing the interaction between HO-3867 and p53 in FT282-CCNE1 cells. Western blot analysis of p53 protein levels in cells treated with DMSO or HO-3867 (50 µM, 3 h) and subjected to increasing temperatures (37 °C to 61 °C). Stabilization of p53 in HO-3867-treated cells indicates compound binding and thermal protection. Vinculin was used as a loading control. **C** Quantification of CETSA results showing the melting temperature (Tₘ) shift of p53 upon HO-3867 treatment. Tₘ was calculated by nonlinear regression curve fitting of normalized p53 band intensities, revealing enhanced thermal stability in HO-3867-treated cells. **D** Western blot analysis showing activation of the p53 signaling pathway in FT282-CCNE1 cells following HO-3867 treatment. Cells were treated with increasing concentrations of HO-3867 for 3 h. Protein levels of phospho-p53 (Ser15), total p53, and the downstream effector p21 were assessed. HO-3867 induced a dose-dependent increase in phospho-p53 and p21 expression, consistent with activation of the p53 pathway. Actin served as the loading control

The tumor suppressor p53, mutated in more than 50% of cancers, is a key driver of chemotherapy resistance and tumor progression, particularly in HGSOCs (Fig. 1A) [5]. Efforts to develop therapeutic agents that reactivate mutant p53 have demonstrated significant promise but face challenges, including off-target toxicity and limited efficacy [8]. Previous studies have shown that HO-3867 selectively induces cancer cell death by converting mutant p53 into a transcriptionally active wild-type-like conformation [14]. In this study, protein–ligand docking analysis revealed that HO-3867 specifically binds to the core domain of mutant p53^Y220C^, providing evidence of a targeted mechanism for p53 reactivation. These findings offer valuable molecular insights into the binding specificity and mode of action of HO-3867, further supporting its potential as a therapeutic agent for cancers harboring mutant p53 (Fig. 3A) [14]. To validate the intracellular interaction between HO-3867 and mutant p53, a cellular thermal shift assay (CETSA) was performed in FT282-CCNE1 cells. The results demonstrated that HO-3867 binds to p53 in vitro, as evidenced by an increase in the melting temperature (Tₘ) of the p53 protein from 44.74 °C to 47.55 °C, indicating enhanced thermal stability upon compound binding (Fig. 3B-C). To further investigate downstream effects, the expression of phospho-p53 (Ser15), total p53, and p21 was assessed following HO-3867 treatment. The results showed that HO-3867 activated p53 signaling, as evidenced by elevated levels of phospho-p53 and p21. Notably, treatment with HO-3867 also led to a reduction in total mutant p53 protein levels (Fig. 3D), suggesting enhanced protein degradation or negative feedback regulation.

**Fig. 4:**
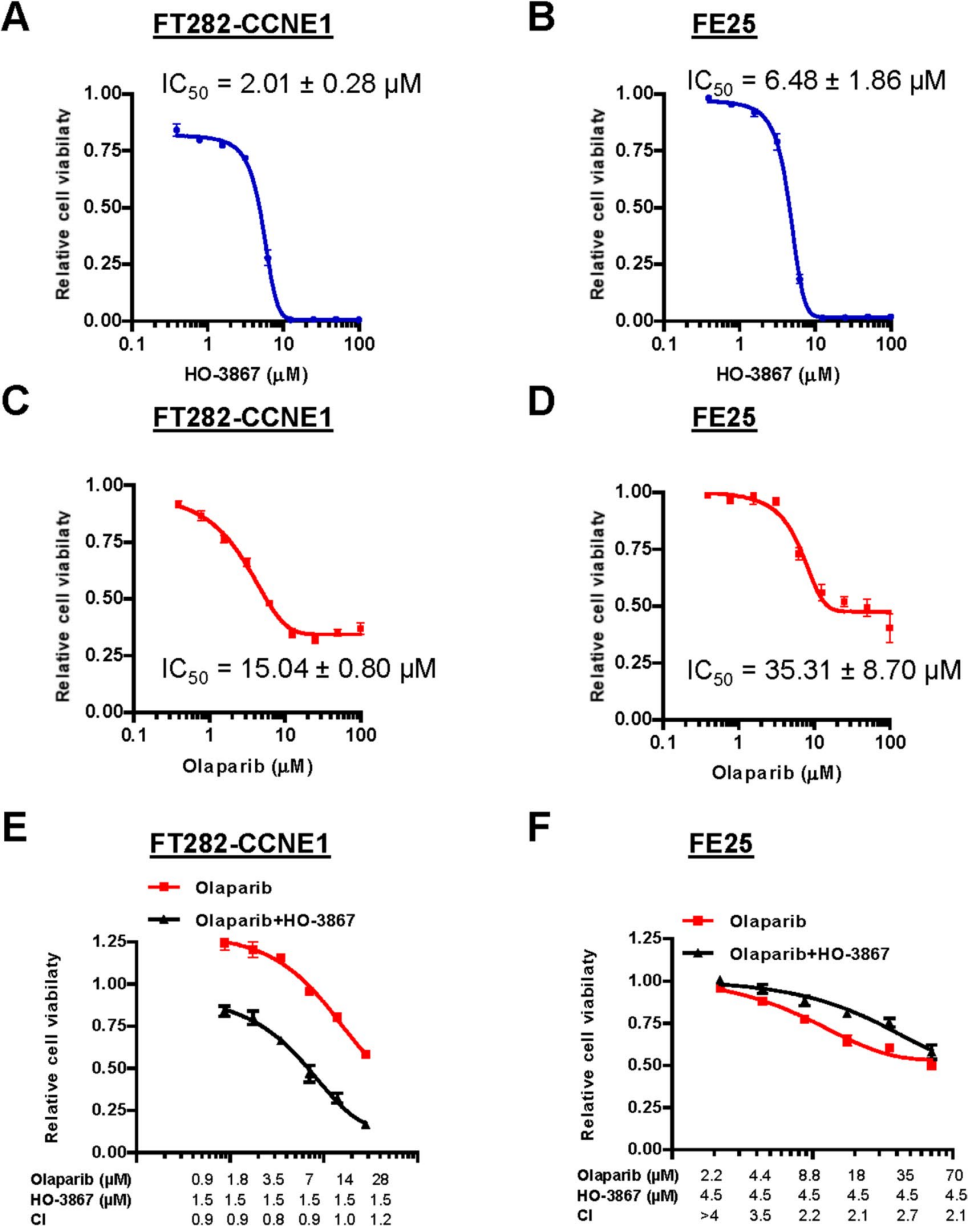
Dose–response analysis of HO-3867, Olaparib, and their combination in FT282-CCNE1 and FE25 cells. **A**, **B** FT282-CCNE1 and FE25 cells were treated with increasing concentrations of HO-3867 for 5 days. **C**, **D** Cells were treated with increasing concentrations of Olaparib for 5 days. **E**, **F** Cells were treated with increasing concentrations of Olaparib in the presence of a fixed concentration of HO-3867 corresponding to its IC₂₅ value. Both drugs were added simultaneously and incubated for 5 days. Cell viability was assessed using the sulforhodamine B (SRB) assay. Each experiment was performed in at least three independent biological replicates, with technical triplicates per condition. Data are presented as mean ± standard error of the mean (S.E.M.) calculated from the biological replicates. Statistical significance was determined by one-way ANOVA followed by Tukey’s post hoc test

Curcumin and its analogs have demonstrated significant anticancer properties, effectively inhibiting various malignancies [51]. Among these compounds, HO-3867 stands out for its selective efficacy against mutant p53. The primary aim of this study was to evaluate the antitumor potential of HO-3867 in human transforming fallopian tube epithelial cell lines, FT282-CCNE1 (p53^R175H^) and FE25 (p53-null). Cytotoxicity was assessed using the sulforhodamine B (SRB) assay. HO-3867 significantly inhibited cell proliferation, with IC₅₀ values ranging from 2.01 µM to 6.48 µM (Fig. 4A–B). FT282-CCNE1 cells, which harbor the p53^R175H^ mutation, were more sensitive to HO-3867 than p53-null FE25 cells. Olaparib exhibited moderate cytotoxicity, with IC₅₀ values between 15.04 µM and 35.31 µM (Fig. 4C–D). Notably, HO-3867 synergistically enhanced the efficacy of Olaparib in FT282-CCNE1 cells (Fig. 4E). Each compound’s half-maximal inhibitory concentration (IC_50_) was determined by analyzing dose–response relationships using CompuSyn software [45]. To evaluate drug synergy, combination treatments were further assessed using the Chou–Talalay method, and combination index (CI) values were calculated at multiple dose levels. CI values less than 1 indicated synergism, and corresponding dose-reduction indices (DRIs) supported enhanced efficacy in the combination treatment. In contrast, this synergistic effect was not observed in p53-null FE25 cells (Fig. 4F), indicating that the interaction is p53-dependent. These results suggest that HO-3867 not only inhibits proliferation in transformed fallopian tube epithelial cells but also enhances the antitumor activity of Olaparib in a manner dependent on mutant p53 expression.

### Targeting TP53 synergies PARP inhibition in Human TFTE cells

**Fig. 5:**
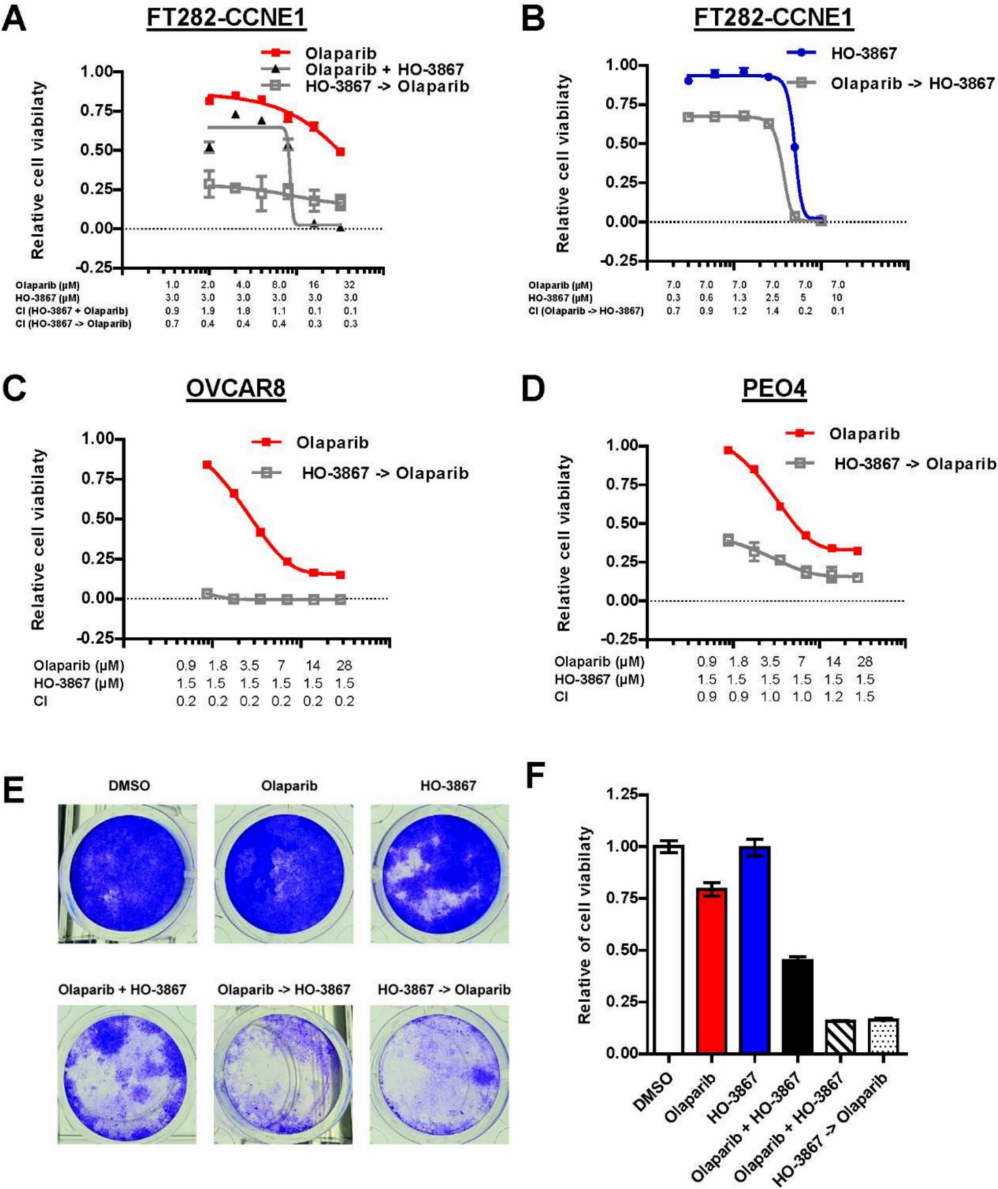
Synergistic effects of HO-3867 and Olaparib on cell viability and colony formation in FT282-CCNE1, OVCAR8, and PEO4 cells. **A** FT282-CCNE1 cells were treated with increasing concentrations of Olaparib with or without a fixed concentration of HO-3867 (3 μM) for 5 days. Combination index (CI) values were calculated using the Chou–Talalay method, where CI < 1 indicates synergy. **B** FT282-CCNE1 cells were treated with increasing concentrations of HO-3867 with or without a fixed concentration of Olaparib (7 μM) for 5 days. **C** OVCAR8 cells were treated with Olaparib, HO-3867, or the combination in a sequential manner (HO-3867 followed by Olaparib), and cell viability was measured after 5 days using the SRB assay. **D** PEO4 cells were treated similarly to (C), with viability assessed after 5 days. **E** FT282-CCNE1 cells were subjected to clonogenic survival assays following treatment with DMSO, Olaparib (16 μM), HO-3867 (4.5 μM), or their combination for 14 days. Colonies were stained with crystal violet. **F** Quantification of colony formation from (E), shown as a percentage relative to the DMSO control. All quantitative data are expressed as mean ± S.E.M. from at least three independent experiments. Statistical analysis was performed using one-way ANOVA followed by Tukey’s post hoc test

In TFTE cells harboring mutant *TP53*, combination treatment with the p53 restorer HO-3867 and the PARP inhibitor Olaparib demonstrated significant anticancer effects. To evaluate the therapeutic potential of combining HO-3867 with Olaparib, we performed SRB assays in FT282-CCNE1, OVCAR8, and PEO4 cells. In FT282-CCNE1 cells, co-treatment with increasing concentrations of Olaparib and a fixed dose of HO-3867 (3 μM) significantly reduced cell viability compared to either agent alone. Combination index (CI) values calculated using the Chou–Talalay method confirmed synergistic effects (CI < 1) (Fig. 5A). Interestingly, reversing the sequence, with Olaparib administered 4 h before HO-3867, Reciprocal treatment with increasing concentrations of HO-3867 and a fixed concentration of Olaparib (7 μM) yielded similar synergy (Fig. 5B). Importantly, sequential administration, whether HO-3867 followed by Olaparib or Olaparib followed by HO-3867, resulted in greater cytotoxicity than simultaneous treatment. These findings suggest that exposing cells to one agent before the other may enhance sensitivity and improve the overall therapeutic effect of the combination.

To validate these findings, the same treatment sequences were tested in a clonogenic formation assay. The combined treatment significantly reduced colony formation compared to either agent alone (Fig. 5E-F), demonstrating its ability to inhibit long-term cancer cell survival. These results underscore the potential of this strategy to overcome challenges such as drug resistance and limited efficacy in *TP53*-mutant cancers. Together, these findings highlight the therapeutic potential of integrating *TP53* modulation with PARPi therapy to improve outcomes in ovarian cancer and other malignancies with defective DNA repair pathways.

### Induction of cell cycle arrest, apoptosis, and DNA damage via combined HO-3867 and olaparib treatment

**Fig. 6:**
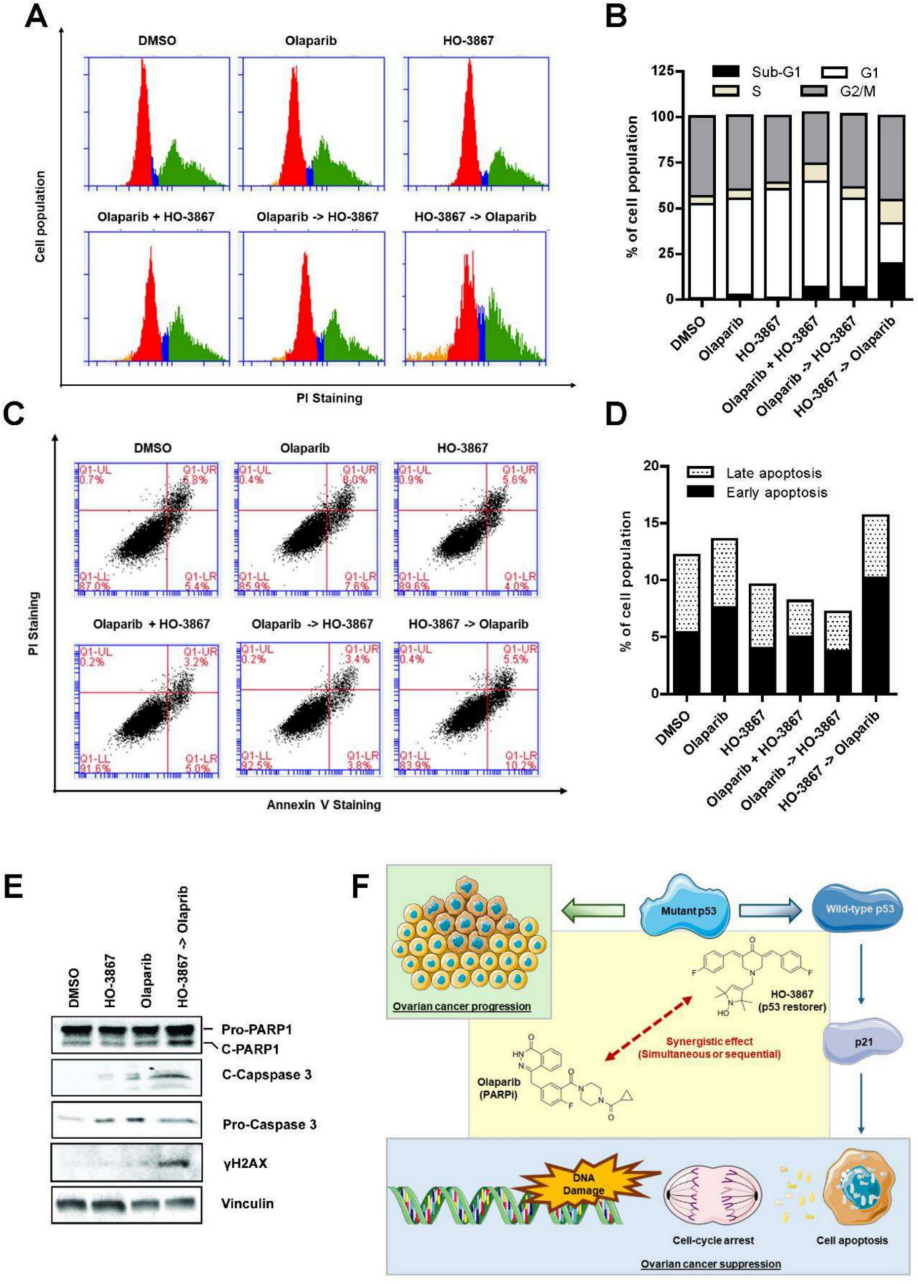
Combination of HO-3867 and Olaparib promotes cell cycle arrest, apoptosis, and DNA damage in FT282-CCNE1 cells. **A** Cell cycle analysis of FT282-CCNE1 cells treated with DMSO, HO-3867 (4.5 μM), Olaparib (16 μM), or their combination for 5 days. In the combination group, the second drug was added 4 h after the first. DNA content was assessed by flow cytometry following PI staining. **B** Quantification of cell cycle phase distribution from (A), showing increased G1 arrest upon combination treatment. **C** Apoptosis analysis using Annexin V/PI staining after treatment as described in **A**. **D** Quantification of apoptotic cells from (C), indicating significantly enhanced apoptosis in the combination group. **E** Western blot analysis of DNA damage and apoptotic markers, including γH2AX, pro- and cleaved forms of PARP1 and caspase-3, in cells treated for 5 days. Vinculin was used as a loading control. **F** Schematic model summarizing the proposed mechanism: HO-3867 reactivates mutant p53 to its wild-type conformation, enhancing p21 expression and sensitizing cells to Olaparib-induced DNA damage. The combined effect, either simultaneous or sequential, promotes cell cycle arrest, apoptosis, and tumor suppression in ovarian cancer cells with mutant p53. Illustrations were adapted from https://smart.servier.com/

The combined treatment of HO-3867 and Olaparib significantly suppressed cell viability in FT282-CCNE1 cells, indicating disruption of cell cycle progression. To investigate further, we analyzed the cell cycle distribution using flow cytometry (FACS) with propidium iodide staining. The simultaneous treatment group exhibited subG1 and G1-phase arrest, suggesting an early apoptotic response. In contrast, cells treated with Olaparib 4 h prior to HO-3867 displayed a delay in subG1 and G2/M phases after 5 days, indicating enhanced DNA damage and disrupted mitotic progression. Notably, pre-treatment with HO-3867 4 h before Olaparib resulted in a pronounced accumulation in the subG1 phase after 5 days, consistent with heightened apoptotic activity. These findings demonstrate that the sequence of drug administration impacts cell cycle disruption and apoptosis, with HO-3867 pre-treatment achieving the most pronounced subG1 accumulation (Fig. 6A-B).

The treatment induced apoptosis, as evidenced by a significant increase in the sub-G1 cell population, a hallmark of apoptotic cell death. Annexin V staining further confirmed a substantial increase in apoptotic cells, with the sequence of administering HO-3867 4 h before Olaparib resulting in the highest apoptotic cell population (Fig. 6C-D). Unexpectedly, several treatment groups, including HO-3867 alone and some combinations, showed slightly lower apoptotic levels compared to the DMSO control. This may be attributed to temporal variation in apoptosis induction or technical variability in staining and detection at this single time point. Additional time-course studies will be needed to better characterize dynamic apoptotic responses. Increased levels of cleaved caspase 3 corroborated this and cleaved PARP1 (Fig. 6E), key markers of apoptosis. Mechanistically, HO-3867, a small molecule with potent anticancer properties, restores mutant p53 function by selectively targeting cancer cells with high therapeutic potential and inducing extensive DNA damage. When combined with the PARP inhibitor Olaparib, the treatment synergistically amplified DNA damage, as demonstrated by elevated γH2AX levels (Fig. 6E), indicative of enhanced DNA damage signaling. This amplified DNA damage likely underpins the observed cell cycle arrest and apoptosis. In summary, this combination therapy induces significant cell cycle arrest, robust apoptosis, and persistent DNA damage, presenting a promising therapeutic strategy for overcoming drug resistance and improving outcomes in cancers with defective DNA repair mechanisms.

## Discussion

The findings from this study highlight the therapeutic potential of HO-3867 as a novel agent for targeting mutant p53-driven ovarian cancers. By reactivating mutant p53 and initiating a wild-type p53-like response, HO-3867 addresses a critical challenge in cancer treatment: overcoming the oncogenic effects of p53 mutations, which are prevalent in over 50% of cancers. Importantly, HO-3867 demonstrated selective cytotoxicity toward cancer cells while sparing normal cells, a characteristic that underscores its favorable safety profile compared to traditional therapies (Fig. 6F).

Mutations in *TP53* impair the tumor-suppressive functions of the p53 protein, disrupting its ability to regulate cell proliferation and apoptosis and driving cancer progression [52]. While these mutations promote tumorigenesis, recent evidence suggests that *TP53* expression is essential for malignant transformation under oncogenic stress, highlighting its complex and context-dependent roles in cancer biology [52]. This duality underscores the need for further investigation into *TP53*’s molecular and clinical implications to improve patient stratification and therapeutic strategies [53]. Our data show that HO-3867 addresses this critical challenge by reactivating mutant p53 and restoring its tumor-suppressive functions, effectively overcoming the oncogenic effects of *TP53* mutations. Notably, HO-3867 demonstrated selective cytotoxicity toward cancer cells while sparing normal cells, offering a favorable safety profile compared to traditional therapies. In comparison to other p53 inhibitors or restorers and MDM2 inhibitors (such as RG7112, RG7388, AM232, SAR405838, NVP-CGM097) [48], HO-3867 exhibits unique efficacy in selectively targeting mutant p53, making it a promising addition to the growing arsenal of p53-targeted therapies aimed at improving outcomes in cancers characterized by *TP53* mutations.

The timing and sequence of drug administration play a pivotal role in determining the efficacy of combination therapies in cancer treatment [54]. Our data reveal that simultaneous administration of HO-3867 and Olaparib results in limited synergy, suggesting that concurrent exposure may not fully exploit the complementary mechanisms of these agents. In contrast, pre-treating cells with HO-3867 4 h before Olaparib leads to the best therapeutic synergy, likely due to HO-3867’s ability to reactivate mutant p53, thereby enhancing the subsequent DNA damage inflicted by Olaparib. Interestingly, administering Olaparib 4 h prior to HO-3867 also enhances synergy, albeit to a lesser degree, suggesting that initial inhibition of PARP and the accumulation of DNA damage may partially prime cancer cells for the effects of HO-3867 (Figs. 5 and 6). It is worth noting that apoptosis levels following HO-3867 or combination treatment appeared lower than in the DMSO control at the 24-h time point. This unexpected trend may be due to technical variability in Annexin V staining or timing-dependent differences in apoptosis induction. Future time-course analyses will be necessary to capture the dynamic apoptotic response more accurately (Figs. 6C-D). These findings emphasize optimizing drug scheduling to maximize their combined efficacy. Sequential drug administration tailored to exploit specific cellular vulnerabilities appears critical for achieving robust therapeutic outcomes. Future studies should further investigate the molecular basis of these timing-dependent effects to refine combination strategies in clinical settings.

Our docking results are consistent with previously published NMR-based findings [14], which identified a conserved binding pocket for HO-3867 on mutant p53. Due to the structural instability and limited crystallizability of the p53^R175H^ mutant, which results from disrupted zinc coordination and folding of the DNA-binding domain, we used the best available wild-type p53 structure for docking analysis. This approach, along with its limitations, is acknowledged and justified given the lack of high-resolution structural data for p53^R175H^ [55]. Despite these promising findings, several critical questions remain unanswered, highlighting the need for further research to unlock the therapeutic potential of HO-3867. First, the precise molecular interactions between HO-3867 and mutant p53 require in-depth investigation to identify specific binding sites and conformational changes that lead to p53 reactivation. Structural studies, such as X-ray crystallography or cryo-electron microscopy, could provide valuable insights into these interactions and aid in optimizing the design of next-generation HO-3867 derivatives with enhanced specificity and potency. Second, although HO-3867 showed greater efficacy in p53-mutant cells, partial growth inhibition was also observed in p53-null FE25 cells. This suggests that HO-3867 may exert additional p53-independent effects. Previous studies have implicated alternative mechanisms, including the modulation of reactive oxygen species (ROS), inhibition of STAT3 signaling, and mitochondrial disruption [56, 57]. HO-3867 selectively inhibits STAT3 activity, induces apoptosis, and exhibits minimal toxicity toward normal cells. Beyond targeting STAT3, HO-3867 downregulates fatty acid synthase (FAS) and focal adhesion kinase (FAK), both critical regulators of cancer cell migration and invasion [58]. Its antitumor efficacy has been demonstrated in ovarian, breast, and pancreatic cancer, and oral squamous cell carcinoma cell lines, both as a monotherapy and in combination with chemotherapeutic agents such as cisplatin and doxorubicin [56, 58–67]. Additionally, HO-3867 activates PTEN in human smooth muscle cells and various tissues, further supporting its broad therapeutic potential [68, 69]. Further investigation is warranted to clarify these p53-independent pathways upon HO-3867 treatment.

To validate the broader applicability of HO-3867 beyond a single p53 mutant context, we extended our investigation to two additional ovarian cancer cell lines, OVCAR8 and PEO4, which harbor distinct p53 mutations. OVCAR8 carries a splice site mutation that results in a Y126 to K132 deletion, while PEO4 contains a G244D missense mutation. In both models, HO-3867 effectively reduced cell viability and showed synergy with Olaparib (Figure), consistent with findings in FT282 CCNE1 cells carrying the R175H mutation. These results support the potential of HO-3867 to exert therapeutic effects across multiple p53 mutant backgrounds. Furthermore, we observed that treatment sequence influenced efficacy, with greater cytotoxicity occurring when Olaparib was administered after HO-3867. Although the precise molecular mechanism remains unclear, we propose that HO-3867 may sensitize cells by reactivating mutant p53 and priming them for DNA damage induced by PARP inhibition. Additional studies involving time course analysis of DNA damage and apoptotic signaling will be necessary to define the basis of this sequence-dependent synergy and inform future therapeutic strategies. Additionally, the broader applicability of HO-3867 in combination therapies warrants exploration. While the current study demonstrates the synergistic effects of HO-3867 with PARP inhibitors like Olaparib, future studies should examine its interactions with other therapeutic modalities, such as immunotherapies, chemotherapies, or targeted therapies. For instance, combining HO-3867 with immune checkpoint inhibitors could potentially modulate the tumor microenvironment by restoring p53 function, thereby enhancing immune system recognition and destruction of cancer cells. Similarly, investigating its role in overcoming resistance to DNA-damaging agents or exploiting synthetic lethality in tumors with specific genetic defects could expand its therapeutic utility.

Furthermore, understanding the pharmacokinetics and pharmacodynamics of HO-3867 in vivo is essential for optimizing its clinical translation. Questions regarding its bioavailability, metabolism, and potential off-target effects need to be addressed through preclinical animal models and early-phase clinical trials. Evaluating its efficacy in patient-derived xenograft (PDX) models and organoid systems could provide insights into its therapeutic potential across heterogeneous tumor profiles. Another critical consideration is translating these preclinical findings into clinical settings. Challenges such as bioavailability, pharmacokinetics, and potential off-target effects must be addressed through rigorous preclinical and clinical trials [70]. Future studies should also explore the drug’s efficacy in patient-derived xenograft (PDX) models to mimic the clinical environment better [71]. Overcoming these translational challenges is critical to bridging the gap between preclinical findings and clinical applications, ultimately paving the way for effective integration into cancer therapy.

Recent molecular studies in preclinical models of colorectal cancer have revealed that sensitivity to PARP inhibitors (PARPi), such as Olaparib, extends beyond homologous recombination deficiency (HRD), highlighting the prospective applications in cancer therapy [72]. Combining Olaparib with innovative agents like HO-3867, a p53 restorer, further enhances therapeutic outcomes by leveraging complementary mechanisms of action. HO-3867 reactivates mutant p53 through covalent binding, triggering a wild-type p53-like anticancer response characterized by selective cytotoxicity toward cancer cells. Since mutant p53 is prevalent in many cancers, including colorectal cancer, its reactivation by HO-3867 can amplify DNA damage and apoptosis when paired with PARPi, even in HR-proficient tumors. This synergistic combination addresses the limitations of PARPi monotherapy, which traditionally relies on HRD-associated biomarkers, and expands its efficacy to a broader patient population [73]. These findings underscore the potential of integrating p53-targeted therapies like HO-3867 with PARPi to overcome resistance mechanisms and improve treatment outcomes in cancers characterized by mutant p53 and replication stress.

Further structure–activity relationship (SAR) studies on HO-3867 and curcumin analogs hold significant potential for enhancing their ability to restore p53 function. By systematically modifying the molecular structure of HO-3867, such as optimizing the diarylidenyl-piperidone (DAP) backbone or the N-hydroxypyrroline (–NOH) group, researchers can explore key interactions with mutant p53 that drive reactivation. Computational modeling and docking studies, combined with biophysical techniques like X-ray crystallography, could identify the structural features critical for binding specificity and stability. Additionally, introducing functional groups with improved bioavailability, metabolic stability, or reduced off-target effects could enhance the therapeutic index of these compounds. For curcumin, known for its pleiotropic anticancer properties but limited pharmacokinetic profile. SAR studies could focus on increasing chemical stability and cellular uptake while retaining its p53-modulating capabilities. Hybridizing structural elements of curcumin and HO-3867 may also create novel derivatives with synergistic effects, leveraging curcumin’s broad-spectrum activity and HO-3867’s targeted p53 reactivation. These rational modifications, guided by SAR insights, have the potential to yield next-generation compounds with superior efficacy and specificity for restoring p53 in cancers harboring *TP53* mutations.

Finally, studies assessing the safety profile of HO-3867 in combination regimens are crucial to ensuring minimal toxicity to normal tissues while maximizing its anticancer effects. Identifying biomarkers that predict responsiveness to HO-3867 could also improve patient stratification and enable personalized therapeutic approaches. Together, these investigations will provide a comprehensive understanding of HO-3867’s capabilities and limitations, paving the way for its successful integration into clinical oncology.

In conclusion, HO-3867 represents a significant advance in developing mutant p53-targeting therapies. Its unique mechanism of action and selective efficacy provide a strong foundation for further investigation, offering hope for improved outcomes in cancers where mutant p53 has been a historically tricky target.

## Data Availability

No datasets were generated or analysed during the current study.
